# Association Between State Hepatitis A Vaccination Requirements and Hepatitis A Vaccination Rates

**DOI:** 10.1093/jpids/piac013

**Published:** 2022-04-06

**Authors:** Yoonyoung Choi, Alexandra Bhatti, Zhiwen Liu, Alex Ruch, Ava Skolnik, Cristina Carias, Michelle G Goveia, Jakub K Simon

**Affiliations:** Center for Observational and Real-world Evidence, Merck & Co., Inc., Kenilworth, New Jersey, USA; Global Vaccine Public Policy and Partnerships, Merck & Co., Inc., Kenilworth, New Jersey, USA; Center for Observational and Real-world Evidence, Merck & Co., Inc., Kenilworth, New Jersey, USA; Center for Observational and Real-world Evidence, Merck & Co., Inc., Kenilworth, New Jersey, USA; Global Vaccine Public Policy and Partnerships, Merck & Co., Inc., Kenilworth, New Jersey, USA; Center for Observational and Real-world Evidence, Merck & Co., Inc., Kenilworth, New Jersey, USA; Global Medical & Scientific Affairs, Merck & Co., Inc., Kenilworth, New Jersey, USA; Clinical Research, Merck & Co., Inc., Kenilworth, New Jersey, USA

**Keywords:** hepatitis A vaccination rates, National Immunization Survey, state hepatitis A vaccination requirements

## Abstract

Using National Immunization Survey Child and Teen (2008-2017), we associated state vaccination requirements with hepatitis A (Hep A) vaccination rates in children and adolescents. States with school entry or both childcare and school entry requirements were associated with 35%-40% higher Hep A vaccination rates, compared with states without such requirements.

## Teaser

Children and adolescents residing in states with school entry or both childcare and school entry requirements had 35%-40% higher hepatitis A vaccination coverage rates, compared with those residing in states without such requirements.

Hepatitis A (Hep A) is a vaccine-preventable, highly contagious liver infection caused by the Hep A virus [1]. Although routine vaccination has been recommended since 2006, the vaccination rate for Hep A is the lowest among all pediatric and adolescent vaccines [2, 3]. State vaccination requirements for children to be vaccinated against certain communicable diseases before childcare or school attendance (unless the child is exempted or conditionally enrolled) are widely considered to be an effective tool for increasing vaccination rates in children and adolescents [4]. However, comparatively fewer states require Hep A vaccination, and limited information is available regarding the utility of such state policies on Hep A vaccination rates. Thus, we determined the association between the Hep A vaccination rate in states with childcare or school requirements compared with states without such requirements among children and adolescents between 2008 and 2017. These data may be useful to inform policymakers considering childcare or school entry requirements for Hep A vaccination in their state.

## METHODS

We conducted a retrospective, cross-sectional study using the National Immunization Survey (NIS)-Child and Teen datasets [5, 6]. We included survey years between 2008 and 2017 during which Hep A vaccination status, verified by healthcare providers, was available for both children and adolescents. That is, our study population represents a subset of children and adolescents in NIS datasets whose immunization history was collected from their vaccination providers. Information on state childcare and school entry requirements for Hep A vaccination was obtained from the WestlawNext legal database. We excluded states that implemented state requirements in one county (AZ) or did not have a verifiable effective date of implementation (NM, PA, TX, UT, and LA) or a provision specific to Hep A vaccination (DE) between 1995 (when the first Hep A vaccine was licensed) and 2017.

States were classified into four exposure groups: states with (1) childcare entry requirements only (C); (2) school entry requirements only (S); (3) childcare and school entry requirement (C + S); and (4) neither childcare nor school requirements in place during the survey year (Controls) (Supplementary Appendix—Table 1) [7]. Controls included states that had no vaccination requirements as well as control periods when states with S, C, or C + S were yet to implement vaccination requirements.

Outcomes were Hep A vaccination initiation and completion, defined as having received 1 or more, or 2 or more, Hep A-containing vaccines, respectively. Descriptive statistics were used to summarize the characteristics of the study population. We compared mean Hep A vaccination rates in the years before and after the implementation of C, S, or C + S vaccination requirements, using the Chi-square statistic test.

Multivariate survey logistic regression was used to quantify the association between state requirements and individual Hep A initiation and completion rates, adjusting for individual- and state-level predictors and the complex sample design [5, 6]. Individual-level predictors of Hep A vaccination in each survey year included age, gender, race and ethnicity, mother’s education level, family income, and insurance status. State-level predictors in each survey year included the year since the requirement was implemented, stringency of nonmedical exemption (NME) requirements (tier 1—NME not permitted, tier 2—most stringent, tier 3—more stringent, tier 4—less stringent, and tier 5—least stringent), the year Advisory Committee on Immunization Practices (ACIP) recommended introducing vaccination in the state (group 1—recommended to introduce routine vaccination in 1999, group 2—recommended to consider routine vaccination in 1999, and group 3—recommended to introduce routine vaccination in 2006), status as a Universal Purchase (UP) program state (UP, UP-Select, and No program), and history of hepatitis A virus outbreak in the previous year. UP is a state-funded program that purchases all ACIP-recommended vaccines at Centers for Disease Control and Prevention (CDC) discounted prices. Detailed operational definitions are provided in Supplementary Appendix—Methods.

Our base case analysis included both children and adolescents (pooled analysis) to demonstrate the overall impact of childcare or school entry requirements on the vaccination rate. We also conducted stratified analyses for young children and adolescents.

All statistical analyses were conducted using SAS version 9.4. As NIS datasets are public-use data files, individuals in the data are not identifiable nor potentially identifiable. Institutional Review Board review and approval were not applicable for our study.

## RESULTS

A total of 43 states with vaccination requirements were included in the analysis: C (5), S (1), C + S (11), and 26 (Controls). As presented in Table 1, a *majority of children were white (72.5%) and non-Hispanic (79.6%), and they had health insurance (93.9%), family’s income* ≤$75 000 (62.1%), and mothers whose education level was non-college graduate or less (63.9%). A majority resided in states that did not meet ACIP thresholds for Hep A vaccination recommendation or consideration in 1999 (69.6%), did not have UP or UP-Select programs (88.5%), and did not experience Hep A outbreaks (86.9%). Among states with requirements, C + S requirements were implemented the earliest (6.7 years prior to a survey year on average) followed by C (3 years) and S (1.5 years). All states with vaccination requirements allowed NMEs; 59.7% resided in states categorized as having tier 3—more stringent NME requirements.

**Table 1.** Demographic Characteristics of Pooled Participants 2008-2017

Characteristic	Unweighted Sample (N = 274 964)	Weighted Sample (N = 20 950 208)
Hep A vaccination initiation		
Yes	65.1%	62.3%
Hep A vaccination completion		
Yes	47.7%	47.7%
Intervention		
Child only	5.4%	2.9%
School only	0.4%	0.5%
Child and School	17.2%	12.0%
Control—No child or school vaccination policy	76.9%	84.6%
Mean years since requirement (SD)		
Child only	4.0(2.7)	3.0(14.6)
School only	1.5(0.5)	1.5(4.9)
Child and School	7.3(4.8)	6.7(33.1)
Tier		
1—No exemption allowed	0%	0%
2—Most stringent exemption process	1.3%	0.8%
3—More Stringent exemption process	10.0%	9.2%
4—Less stringent exemption process	7.0%	1.8%
5—Least stringent exemption process	4.8%	3.6%
Not applicable (control states)	76.9%	84.6%
State groups based on ACIP recommendation		
Group 1—Routine recommendation since 1999	17.8%	24.2%
Group 2—Consideration of routine recommendation since 1999	11.0%	6.2%
Group 3—Routine recommendation since 2006	71.2%	69.6%
Universal Purchase (UP) status		
UP	12.8%	4.9%
UP-Select	12.0%	6.6%
None	75.2%	88.5%
hepatitis A virus outbreak in the past year		
Yes	6.5%	13.1%
Survey year		
2008	10.4%	10.3%
2009	10.3%	10.3%
2010	10.1%	10.2%
2011	11.1%	9.8%
2012	10.0%	10.1%
2013	9.1%	10.1%
2014	9.6%	9.7%
2015	9.4%	9.3%
2016	9.9%	10.1%
2017	10.0%	10.0%
Age		
19-23 mo	12.9%	6.6%
24-29 mo	14.4%	7.5%
30-35 mo	17.3%	7.9%
13 y	11.2%	15.3%
14 y	11.4%	15.5%
15 y	11.2%	16.3%
16 y	11.3%	16.1%
17 y	10.3%	14.8%
Gender		
Male	51.8%	51.2%
Female	48.2%	48.8%
Race		
White only	77.3%	72.5%
Black only	9.8%	15.4%
Other and multiple race	12.9%	12.1%
Ethnicity		
Hispanic	12.4%	20.4%
Non-Hispanic	87.6%	79.6%
Mother’s education level		
Less than 12 years	9.4%	14.0%
12 years	17.6%	25.0%
More than 12 years, non-college graduate	27.0%	24.9%
College graduate	46.0%	36.1%
Family income		
<$20 001	14.0%	20.0%
$20 001-$40K	15.5%	19.7%
$40 001-$75K	22.4%	22.4%
$75 001+	42.5%	37.9%
Missing	5.7%	
Insurance status		
Private insurance only	55.6%	48.7%
Any Medicaid	28.3%	34.2%
Other insurance	11.5%	11.0%

This table includes all study populations, children and adolescents residing in states with or without childcare or school requirements. The percentages are the proportions of the unweighted sample (exact sample in NIS data) and the weighted sample (multiplied with NIS sampling weights to make the sample nationally representative), meeting each characteristic.

Hep A, Hepatitis A; ACIP, Advisory Committee on Immunization Practices; NIS, National Immunization Survey.

Mean rates of Hep A vaccination initiation and completion increased significantly following the implementation of childcare and school vaccination requirements (Supplementary Appendix—Figure 1). After adjusting for individual- and state-level predictors, children and adolescents (pooled analysis) residing in states with S or C + S requirements had 35%-40% higher odds of initiating or completing Hep A vaccination compared with those residing in control states (Table 2). Individual-level predictors associated with higher Hep A vaccination initiation or completion rates included recent survey year, being nonwhite, Hispanic, of younger age, having a high family income, insurance, and a college graduate mother. State-level predictors associated with higher Hep A vaccination initiation or completion rates included residing in states with the most stringent NME process, states in which ACIP recommended (or considered) Hep A vaccination since 1999, and states without UP programs.

**Table 2.** Association Between State Policy and Vaccination Initiation and Completion Rates—Multivariate Pooled Analysis

	Initiation		Completion	
	Odds Ratio	Confidence Interval	Odds Ratio	Confidence Interval
Intervention				
Control—No school or child policy	reference			
Child only	1.15	(0.96, 1.36)	1.17	(0.99, 1.39)
School only	1.35	(1.10, 1.66)^*^	1.36	(1.12, 1.65)^*^
Child and School	1.37	(1.05, 1.78)^*^	1.4	(1.09, 1.79)^*^
Tier				
5—Least stringent exemption process	reference			
4—Less stringent exemption process	1.29	(0.99, 1.69)	1.11	(0.87, 1.42)
3—More stringent exemption process	1.16	(0.92, 1.47)	1.08	(0.86, 1.34)
2—Most stringent exemption process	1.61	(1.20, 2.15)^*^	1.17	(0.90, 1.53)
State groups based on ACIP recommendation				
Group 3—Routine recommendation since 2006	reference			
Group 2—Consideration of routine recommendation since 1999	1.31	(1.15, 1.48)^*^	1.2	(1.06, 1.37)^*^
Group 1—Routine recommendation since 1999	3.65	(3.18, 4.19)^*^	2.85	(2.50, 3.25)^*^
Universal Purchase (UP) or UP-Select states				
Neither	reference			
UP	0.95	(0.85, 1.07)	0.94	(0.84, 1.05)
UP-Select	0.89	(0.80, 0.98)^*^	0.9	(0.82, 0.98)^*^
hepatitis A virus outbreak in the past year				
No	reference			
Yes	1.02	(0.94, 1.11)	1.04	(0.97, 1.12)
Survey year				
2008	reference			
2009	1.33	(1.23, 1.43)^*^	1.35	(1.24, 1.47)^*^
2010	1.63	(1.51, 1.76)^*^	1.72	(1.58, 1.88)^*^
2011	2.1	(1.95, 2.27)^*^	2.18	(2.00, 2.37)^*^
2012	2.57	(2.37, 2.79)^*^	2.69	(2.46, 2.94)^*^
2013	3.02	(2.77, 3.28)^*^	3.1	(2.84, 3.40)^*^
2014	3.63	(3.33, 3.96)^*^	3.93	(3.59, 4.31)^*^
2015	4.27	(3.88, 4.68)^*^	4.42	(4.02, 4.87)^*^
2016	5.11	(4.66, 5.59)^*^	5.21	(4.75, 5.73)^*^
2017	5.86	(5.33, 6.45)^*^	5.94	(5.41, 6.54)^*^
Age				
17 y	reference			
16 y	1.06	(0.99, 1.14)	1.07	(1.00, 1.15)
15 y	1.13	(1.06, 1.21)^*^	1.15	(1.07, 1.23)^*^
14 y	1.24	(1.16, 1.33)^*^	1.25	(1.17, 1.34)^*^
13 y	1.27	(1.19, 1.35)^*^	1.29	(1.20, 1.38)^*^
30-35 mo	4.4	(4.09, 4.73)^*^	2.76	(2.57, 2.95)^*^
24-29 mo	4.29	(3.98, 4.62)^*^	1.94	(1.81, 2.09)^*^
19-23 mo	2.93	(2.72, 3.15)^*^	0.5	(0.46, 0.54)^*^
Gender				
Male	reference			
Female	1.01	(0.98, 1.05)	1.03	(0.99, 1.07)
Race				
White only	reference			
Black only	1.42	(1.34, 1.50)^*^	1.27	(1.20, 1.34)^*^
Other and multiple race	1.42	(1.33, 1.53)^*^	1.29	(1.21, 1.37)^*^
Ethnicity				
Non-Hispanic	reference			
Hispanic	1.67	(1.56, 1.78)^*^	1.55	(1.46, 1.64)^*^
Mother’s education level				
Less than 12 years	reference			
12 years	0.9	(0.84, 0.97)^*^	0.91	(0.85, 0.98)^*^
More than 12 years, non-college graduate	0.92	(0.85, 0.99)^*^	0.95	(0.89, 1.02)
College graduate	1.14	(1.05, 1.23)^*^	1.19	(1.10, 1.28)^*^
Family income				
<$20 000	reference			
$20 001-$40K	0.92	(0.86, 0.98)^*^	0.96	(0.90, 1.03)
$40 001-$75K	0.85	(0.79, 0.91)^*^	0.92	(0.86, 0.99)^*^
$75 001+	1.06	(0.99, 1.15)	1.13	(1.05, 1.22)^*^
Insurance status				
Uninsured	reference			
Private insurance only	1.31	(1.19, 1.44)^*^	1.38	(1.26, 1.51)^*^
Any Medicaid	1.65	(1.51, 1.82)^*^	1.63	(1.48, 1.78)^*^
Other insurance	1.51	(1.36, 1.67)^*^	1.56	(1.41, 1.73)^*^

State indicators were adjusted in a final model, but the results were not reported for simplicity.

ACIP, Advisory Committee on Immunization Practices.

*P* < .05.

The stratified analyses revealed that this result was driven by 36%-73% higher vaccination rates in adolescents residing in states with C, S, or C + S requirements (Supplementary Appendix—Table 2). Among children, only S requirements were associated with 36%-55% higher Hep A vaccination rates.

## DISCUSSION

Using NIS datasets 2008-2017, we found that children and adolescents residing in states with S or C + S requirements were more likely to initiate and complete Hep A vaccination, compared with those residing in states without such requirements. Our findings are similar to other studies that have evaluated the impact of other school vaccination requirements on vaccination rates [8].

Our results suggest that the implementation of a Hep A vaccination requirement and other policies that impact adherences to the requirements are also important to influence the Hep A vaccination rate. In this study, more stringent NMEs were associated with higher Hep A vaccination rates. Of note, Omer et al reported a lower incidence of pertussis in states with more difficult vaccination exemption procedures [9]. Additionally, our study did now show a positive association between UP or UP-Select status and Hep A vaccination rates, unlike Freed et al who had shown that the implementation of a UP program was associated with improved immunization rates among children under 2 years of age [10].

Different factors may explain why the association of state requirements on Hep A vaccination rates was more pronounced among adolescents than children, namely (1) higher baseline vaccination rates in children than adolescents and (2) less universal attendance to childcare than school (64% of eligible children attend childcare, while >90% of eligible children attend school) [11]. Thus, higher Hep A vaccination rates in young children in states with school entry requirements may reflect provider’s or parents’ anticipation of school entry.

Our study has several strengths and limitations. The main strength of our study was the ability to consider several individual- and state-level predictors for Hep A vaccination and controls to help balance state-specific or period-specific propensity for Hep A vaccination. Also, we used provider-verified vaccination data to avoid inaccurate recollection and social desirability bias. However, the study has several limitations. Due to the cross-sectional nature of this study, some of captured Hep A vaccination could occur before such statewide events (ie, UP/UP-Select and outbreaks). In addition, our outcome variables in NIS datasets represented the number of Hep A vaccinations cumulated by the time of interview (and by 36 months of age for NIS-Child). While those affect both intervention and control states, the effects of state requirements could be inflated by classifying vaccination occurring in pre-periods as that occurring in post-periods. Finally, due to the population included in NIS datasets, we could not include the entire population eligible for childcare or school entry, possibly attenuating true policy effects.

## CONCLUSION

Children and adolescents residing in states with school or childcare and school vaccination requirements were found to have higher Hep A initiation and completion rates compared with those residing in states without such policies. The stratified analyses revealed that this result was driven by increased vaccination rates in adolescents residing in states with C, S, or C + S. The results can inform policymakers considering Hep A vaccination requirements to improve pediatric and adolescent Hep A vaccination coverage rates.

## Supplementary Material

piac013_suppl_Supplementary_Material

## Notes

***Author contributions***. Y. C. conceptualized and designed the study, coordinated and supervised data analyses, contributed to interpretation of data and analyzed results, and drafted the initial manuscript. A. B. conceptualized and designed the study, contributed to interpretation of data and analyzed results, and drafted the initial manuscript. Z. L. designed the study, coordinated and supervised data analyses, and contributed to interpretation of data and analyzed results. M. G. G. and J. K. S. designed the study and contributed to interpretation of data and analyzed results. C. C., A. R., and A. S. contributed to interpretation of data and analyzed results. All authors critically reviewed the manuscript for important intellectual content, revised the manuscript, and approved the final manuscript as submitted, and they also agreed to be accountable for all aspects of the work.

***Financial support***. This study was supported by Merck Sharp & Dohme Corp., a subsidiary of Merck & Co., Inc., Kenilworth, NJ, USA. The funder/sponsor did not participate in the work.

***Potential conflicts of interest***. Y. C. and other contributing authors are or were (Z. L. and J. S.) employed by Merck Sharp & Dohme Corp., a subsidiary of Merck & Co., Inc., Kenilworth, NJ, USA, at the time of the study. Current affiliations of Z. L. and J. S. are Johnson & Johnson., Inc., New Brunswick, New Jersey, USA, and Orel Consulting., LLC., Doylestown, Pennsylvania, USA, respectively.
