# Supplementary material for: Association Between State Hepatitis A Vaccination Requirements and Hepatitis A Vaccination Rates
Source: J Pediatric Infect Dis Soc. 2022 Apr 6;11(6):295–9. doi: 10.1093/jpids/piac013 (PMC9214775; doi:10.1093/jpids/piac013)
Supplement: piac013_suppl_Supplementary_Material [file piac013_suppl_supplementary_material.docx]

**Appendix**

**Table 1. Classification of states according to state requirement types**

| # | State | Type of requirement | Year of implementation | Stringency of requirement* |
| --- | --- | --- | --- | --- |
| 1 | AK | C+S | S: 2001-Jul-1  C: 2001-Jul-1 | Both: Tier 3 |
| 2 | AR | C+S | S: 2014-Sept  C: 2014-Sept | Both: Tier 2 |
| 3 | CT | C+S | S: 2011-Aug 1  C: 2011-Aug 1 | Both: Tier 3 |
| 4 | DC | C+S | S: 2009-2010 Aug  C: 2009-2010 Aug | Both: Tier 4 |
| 5 | GA | C+S | S: 2007-2008 Aug  C: 2007-2008 Aug | Both: Tier 3 |
| 6 | ID | C+S | S: 2011-April 7  C: 2011-April 7 | Both: Tier 4 |
| 7 | KY | C+S | S: 2018-2019 Jul  C: 2018-Jul-1 | Both: Tier 3 |
| 8 | NV | C+S | S: 2002-2003 Jun  C: 2002-2003 Jun | Both: Tier 5 |
| 9 | OK | C+S | S: 1998-Nov-1  C: 1998-Nov-1 | Tier 3 |
| 10 | OR | C+S | S: 2008-09 school year (2008-August)  C: 2008-09 school year (2008-August) | Both: Tier 4 (2008-2014 Mar)  Tier 2 (2014 Mar-) |
| 11 | TN | C+S | S: 2011-Jul-1  C: 2010-Jul-1 | Both: Tier 3 |
| 12 | KS | C | C: 2009 Aug | Tier 5 |
| 13 | ND | C | C: 2007-Aug-1 | Tier 4 |
| 14 | OH | C | C: 2015-Mar-19 | Tier 5 |
| 15 | MN | C | C: 2014 Sept 1 | Tier 3 |
| 16 | RI | C | C: 2015 -Aug-1 | Tier 4 |
| 17 | IN | S | S: 2015- Jul-1 | Tier 5 |
| 18 | AL, CA, CO,  FL, HI, IA, IL, MA, MD, ME, MI, MO, MS, MT, NC, NE, NH, NJ, NY, SD, VA, VT, WA, WI, WV, WY | Controls | Not applicable | Not applicable |

Note: C=childcare entry requirements only; S=school entry requirements only; C+S=childcare and school entry requirement; and Controls= neither childcare nor school requirements in place during the survey year.

*Tier1 = NME not permitted; Tier2= most stringent NME; Tier3= more stringent NME; Tier4=less stringent NME; and Tier5=least stringent NME

**Methods. Operational definitions of outcomes and covariates**

***Outcomes***

Hep A vaccination initiation and completion were defined using the P_NUMHEA (NIS-Child) and P_NUMHEPA (NIS-Teen) variables. These variables provide the number of provider-verified hepatitis A-containing vaccines received, excluding any vaccinations after the interview date. Hep A vaccination initiation and completion were defined as having received 1 or more, or 2 or more, Hep A-containing vaccines respectively.

***Individual-level Covariates***

We used survey year, age, race, gender, and mother’s education level as collected in NIS data sets. We modified variables for family income, ethnicity, and insurance status. We categorized family income into ≤ $20,000, $20,001-$40,000, $40,001-$75,000 and ≥ $75,001; grouped ethnicity as Hispanic vs non-Hispanic and insurance status as private insurance only, any Medicaid, other insurance, or uninsured. Other insurance included S-CHIP, TRICARE, or Indian Health Service. When multiple insurance types were selected for a child or adolescent, we applied the following hierarchy: any Medicaid; other insurance; private insurance and uninsured.

***State-level Covariates***

We considered several state-level confounders. The degree to which childcare or school entry requirements can be enforced depends on certain attributes of the law such as what types of exemptions are permitted and under what conditions. We expanded a previous tri-level non-medical exemption (NME) categorization by Omer et al[1] from “easy, medium, difficult” categories to 5 tiers (Tier 1 – NME not permitted, Tier 2 – most stringent, Tier 3- more stringent, Tier 4 – less stringent, Tier 5- least stringent). Tier 1 is the most restrictive since the state law does not permit NMEs. Tier 2 requires an enhanced educational component together with parental acknowledgment of risks associated with non-vaccination, parental signature, documented on a department-approved form, and in one case, notarization of the form. Tier 3 requires the department-approved form to be signed by the parent, and that the form be notarized. Tier 4 requires that the exemption be documented on a department-approved form, signed by the parent and in some cases, the parent must acknowledge risk of non-vaccination. Tier 5 only requires a parental signature, and the law does not expressly indicate that it must be on a department-approved form.

We also grouped states based on the year that ACIP recommended routine Hep A vaccination for children in the state: routine vaccination recommended in 1999 (Group 1); consideration of routine vaccination recommended in 1999 (Group 2); and routine vaccination recommended in 2006 (Group 3) [2]. Second, we considered states’ status as a Universal Purchase (UP) or UP-Select state. Universal Purchase is a state-funded program that purchases all ACIP recommended vaccines at CDC discounted prices. Similarly, UP-Select is a modified version of UP that only includes the purchase of select ACIP-recommended vaccines and has specific participant eligibility criteria per state. UP effectively removes the traditional “buy and bill” process such that healthcare providers no longer need to purchase the vaccine up front and then bill for it to be reimbursed. We determined whether states have a UP or UP-SELECT program according to VFC Childhood Vaccine Supply Policy [3-8]. When information was missing in a survey year, we used the state’s designation in the previous year. Finally, we considered a history of HAV outbreak in states. We defined a history of HAV outbreak as an outbreak in the state that occurred within one year of the current survey year, referring to CDC’s Division of Viral Hepatitis (DVH) reporting [9] as well as the National Outbreak Reporting System (NORS) [10].

**Figure 1. Hep A vaccination initiation and completion rates for children and adolescents by status of state vaccination requirements**

Completion rates

Initiation rates

Note: C=childcare entry requirements only; S=school entry requirements only; C+S=childcare and school entry requirement; and Controls= neither childcare nor school requirements in place during the survey year.

* pre- vs post-periods of requirement implementation.

Every pair of weighted vaccination rates for pre- vs post-periods of requirement implementation were significantly different (p-value <.0001). In this comparison only, we assigned 2008-2012 as pre-periods and 2013-2017 as post-periods for control states.

**Table 2. Multivariate association between state policy and vaccination initiation and completion rate stratified by teen and children**

| **Predictors** | **Teen** | | | | **Child** | | | |
| --- | --- | --- | --- | --- | --- | --- | --- | --- |
|  | **Initiation** | | **Completion** | | **Initiation** | | **Completion** | |
|  | **Odds Ratio** | **Confidence Interval** | **Odds Ratio** | **Confidence Interval** | **Odds Ratio** | **Confidence Interval** | **Odds Ratio** | **Confidence Interval** |
| Intervention | | | | | | | | |
| Control | reference | | | | | | | |
| Child only | 1.22 | (0.99, 1.50) | 1.36 | (1.10, 1.69)* | 1 | (0.74, 1.36) | 0.76 | (0.59, 0.96)* |
| School only | 1.39 | (1.10, 1.77)* | 1.44 | (1.13, 1.84)* | 1.55 | (1.05, 2.29)* | 1.36 | (1.03, 1.80)* |
| Child and School | 1.59 | (1.16, 2.16)* | 1.73 | (1.26, 2.37)* | 0.82 | (0.50, 1.34) | 0.78 | (0.55, 1.12) |
| Tier | | | | | | | | |
| 5 - Least stringent exemption process | Reference | | | | | | | |
| 4 - Less stringent exemption process | 1.11 | (0.81, 1.51) | 0.88 | (0.64, 1.19) | 2.26 | (1.35, 3.80)* | 2.08 | (1.45, 3.00)* |
| 3 - More stringent exemption process | 1.04 | (0.79, 1.37) | 0.99 | (0.75, 1.31) | 1.96 | (1.27, 3.04)* | 1.39 | (1.01, 1.91)* |
| 2 - Most stringent exemption process | 1.45 | (1.02, 2.04)* | 1 | (0.71, 1.41) | 2.9 | (1.69, 4.95)* | 2.04 | (1.39, 3.01)* |
| State groups based on ACIP recommendation | | | | | | | | |
| Group 3 - Universal recommendation since 2006 | Reference | | | | | | | |
| Group 2 - Consideration of routine recommendation since 1999 | 1.7 | (1.45, 2.00)* | 1.65 | (1.39, 1.95)* | 0.45 | (0.38, 0.54)* | 0.43 | (0.36, 0.51)* |
| Group 1 - Routine recommendation since 1999 | 4.69 | (3.96, 5.57)* | 4.29 | (3.60, 5.12)* | 1.15 | (0.95, 1.39) | 0.79 | (0.66, 0.93)* |
| Universal Purchase (UP) status | | | | | | | | |
| Neither | Reference | | | | | | | |
| UP | 0.92 | (0.80, 1.07) | 0.89 | (0.77, 1.03) | 1.13 | (0.96, 1.34) | 1.12 | (0.97, 1.29) |
| UP-Select | 0.88 | (0.78, 1.00)* | 0.84 | (0.75, 0.95)* | 0.94 | (0.82, 1.09) | 1.1 | (0.97, 1.24) |
| HAV outbreak in the past year | | | | | | | | |
| No | Reference | | | | | | | |
| Yes | 1 | (0.91, 1.10) | 1.02 | (0.93, 1.12) | 1.11 | (0.98, 1.26) | 1.09 | (0.99, 1.21) |
| Survey year | | | | | | | | |
| 2008 | Reference | | | | | | | |
| 2009 | 1.38 | (1.25, 1.52)* | 1.39 | (1.24, 1.57)* | 1.25 | (1.14, 1.37)* | 1.33 | (1.21, 1.46)* |
| 2010 | 1.71 | (1.55, 1.89)* | 1.87 | (1.66, 2.11)* | 1.52 | (1.37, 1.68)* | 1.55 | (1.40, 1.71)* |
| 2011 | 2.29 | (2.08, 2.52)* | 2.49 | (2.22, 2.80)* | 1.75 | (1.59, 1.94)* | 1.73 | (1.57, 1.91)* |
| 2012 | 2.88 | (2.60, 3.20)* | 3.28 | (2.91, 3.70)* | 1.92 | (1.72, 2.15)* | 1.78 | (1.61, 1.98)* |
| 2013 | 3.45 | (3.11, 3.83)* | 3.87 | (3.43, 4.36)* | 2.06 | (1.82, 2.33)* | 1.93 | (1.73, 2.15)* |
| 2014 | 4.16 | (3.74, 4.63)* | 5.08 | (4.50, 5.73)* | 2.49 | (2.19, 2.84)* | 2.22 | (1.98, 2.49)* |
| 2015 | 5 | (4.46, 5.61)* | 5.75 | (5.06, 6.52)* | 2.52 | (2.20, 2.88)* | 2.4 | (2.14, 2.69)* |
| 2016 | 6.24 | (5.59, 6.96)* | 7.07 | (6.24, 8.01)* | 2.42 | (2.10, 2.78)* | 2.47 | (2.20, 2.77)* |
| 2017 | 7.31 | (6.51, 8.22)* | 8.54 | (7.52, 9.70)* | 2.49 | (2.17, 2.86)* | 2.34 | (2.09, 2.63)* |
| Age – Teen, Children |  |  |  |  |  |  |  |  |
| 17 years, 30-35 months | Reference | | | | | | | |
| 16 years | 1.07 | (0.99, 1.15) | 1.08 | (1.00, 1.16) | NA | | | |
| 15 years | 1.14 | (1.06, 1.22)* | 1.15 | (1.07, 1.24)* |  |  |  |  |
| 14 years, 24-29 months | 1.25 | (1.17, 1.34)* | 1.27 | (1.18, 1.37)* | 0.98 | (0.91, 1.05) | 0.74 | (0.69, 0.78)* |
| 13 years, 19-23 months | 1.28 | (1.19, 1.37)* | 1.31 | (1.22, 1.40)* | 0.69 | (0.64, 0.74)* | 0.22 | (0.21, 0.24)* |
| Gender | | | | | | | | |
| Male | Reference | | | | | | | |
| Female | 1.02 | (0.97, 1.06) | 1.03 | (0.99, 1.08) | 1 | (0.95, 1.06) | 1.02 | (0.97, 1.07) |
| Race | | | | | | | | |
| White only | Reference | | | | | | | |
| Black only | 1.47 | (1.37, 1.57)* | 1.36 | (1.27, 1.46)* | 1.25 | (1.15, 1.37)* | 1.01 | (0.93, 1.09) |
| Other and Multiple race | 1.47 | (1.35, 1.60)* | 1.38 | (1.27, 1.50)* | 1.31 | (1.19, 1.44)* | 1.11 | (1.03, 1.20)* |
| Ethnicity | | | | | | | | |
| Non-Hispanic | Reference | | | | | | | |
| Hispanic | 1.74 | (1.61, 1.89)* | 1.68 | (1.55, 1.82)* | 1.44 | (1.32, 1.58)* | 1.28 | (1.19, 1.38)* |
| Mother's education level | | | | | | | | |
| Less than 12 years | Reference | | | | | | | |
| 12 years | 0.87 | (0.79, 0.96)* | 0.87 | (0.79, 0.96)* | 1.03 | (0.93, 1.15) | 1.07 | (0.98, 1.18) |
| More than 12 years, non-college grad | 0.88 | (0.81, 0.97)* | 0.91 | (0.83, 1.00) | 1.11 | (1.00, 1.23) | 1.14 | (1.04, 1.25)* |
| College graduate | 1.09 | (0.99, 1.20) | 1.14 | (1.04, 1.26)* | 1.34 | (1.19, 1.50)* | 1.32 | (1.20, 1.46)* |
| Family income | | | | | | | | |
| <$20001 | Reference | | | | | | | |
| $20001-$40K | 0.91 | (0.83, 0.98)* | 0.95 | (0.87, 1.03) | 0.92 | (0.84, 1.01) | 0.98 | (0.90, 1.06) |
| $40001-$75K | 0.83 | (0.76, 0.90)* | 0.89 | (0.82, 0.98)* | 0.92 | (0.83, 1.02) | 0.99 | (0.91, 1.09) |
| $75001+ | 1.09 | (1.00, 1.19) | 1.17 | (1.06, 1.28)* | 0.95 | (0.85, 1.07) | 1.05 | (0.95, 1.16) |
| Insurance status | | | | | | | | |
| Uninsured | Reference | | | | | | | |
| Private insurance only | 1.24 | (1.11, 1.39)* | 1.37 | (1.22, 1.54)* | 1.88 | (1.65, 2.15)* | 1.75 | (1.54, 1.98)* |
| Any Medicaid | 1.63 | (1.46, 1.82)* | 1.7 | (1.51, 1.91)* | 2.01 | (1.78, 2.28)* | 1.74 | (1.54, 1.96)* |
| Other insurance | 1.46 | (1.29, 1.65)* | 1.62 | (1.42, 1.84)* | 1.94 | (1.68, 2.24)* | 1.63 | (1.42, 1.88)* |

Note: * p<0.05. State indicators were adjusted in a final model, but the results were not reported for simplicity. NA=not appliable

**References**

1. Omer, S.B., et al., *Nonmedical Exemptions to School Immunization RequirementsSecular Trends and Association of State Policies With Pertussis Incidence.* JAMA, 2006. **296**(14): p. 1757-1763.
2. Nelson, N.P., et al., *Hepatitis A vaccination coverage among adolescents (13-17 years) in the United States, 2008-2016.* Vaccine. 2018 Mar 14;**36**(12):1650-1659.
3. Association of Immunization Managers. *Childhood Vaccine Financing and Supply Policy for Private Privders Map* 2014; Available from: <https://cdn.ymaws.com/www.immunizationmanagers.org/resource/resmgr/Private_Provider_Vax_Supply_.pdf>.
4. Association of Immunization Managers. *Immunization Program Grantees’ Childhood Vaccine Financing / Supply Policy for Private Providers (as of January 2011)*2011.
5. National Center for Immunization and Respiratory Diseases. *Vaccines for Children Program (VFC)*. 2009 June, 9 2009; Available from: <https://www.cdc.gov/vaccines/programs/vfc/about/vac-supply-policy/supply-2009.html>.
6. National Center for Immunization and Respiratory Diseases. *Vaccines for Children Program (VFC)*. 2008 October 22, 2010; Available from: <https://www.cdc.gov/vaccines/programs/vfc/about/vac-supply-policy/supply-2008.html>.
7. Association of Immunization Managers. *Childhood Vaccine Financing and Supply Policy for Private Privders Map* 2015 Nov. 2015; Available from: <https://cdn.ymaws.com/www.immunizationmanagers.org/resource/resmgr/Policy_Maps/Vax_Finance_private_4-21-16.pdf>.
8. Association of Immunization Managers. *Childhood Vaccine Financing and Supply Policy for Private Privders Map* 2016 March 2017; Available from: <https://cdn.ymaws.com/aim.site-ym.com/resource/collection/5E0587B6-CC4A-4A7B-999C-0C1EE0855BA6/AIM%20Policy%20Maps%202016%204-4-17vax%20fin%20private.pdf>.
9. Centers for Disease Control and Prevention. *National Outbreak Reporting System (NORS): Outbreaks per State*. 2018 December 7, 2018 Available from: <https://wwwn.cdc.gov/norsdashboard/>.
10. Centers for Disease Control and Prevention. Widespread person-to-person outbreaks of heapatitis A across the United States. 2019 [cited 2019 November 4, 2019]; Available from: <https://www.cdc.gov/hepatitis/outbreaks/2017March-HepatitisA.htm>.
